# Evaluation of circulating tumor DNA as a prognostic biomarker for metastatic pancreatic adenocarcinoma

**DOI:** 10.3389/fonc.2022.926260

**Published:** 2022-08-23

**Authors:** Shasha Guan, Guochao Deng, Jingjie Sun, Quanli Han, Yao Lv, Tianhui Xue, Lijuan Ding, Tongxin Yang, Niansong Qian, Guanghai Dai

**Affiliations:** ^1^ Department of Oncology, Hainan Hospital of Chinese People’s Liberation Army (PLA) General Hospital, Sanya, China; ^2^ Senior Department of Oncology, The Fifth Medical Center of Chinese People’s Liberation Army (PLA) General Hospital, Beijing, China; ^3^ Department of Thoracic Oncology, The Eighth Medical Center, Chinese People’ Liberation Army (PLA) General Hospital, Beijing, China; ^4^ The Second School of Clinical Medicine, Southern Medical University, Guangzhou, China

**Keywords:** circulating tumor DNA (ctDNA), pancreatic adenocarcinoma (PAC), prognosis, KRAS, TP53, CDKN2A, SMAD4

## Abstract

**Purpose:**

Pancreatic cancer is an aggressive solid tumor with a severe prognosis. Although tumor biomarkers are often used to identify advanced pancreatic cancer, this is not accurate, and the currently used biomarkers are not indicative of prognosis. The present study evaluated circulating tumor DNA (ctDNA) as a biomarker for prognosis prediction and disease monitoring in metastatic pancreatic adenocarcinoma (PAC).

**Methods:**

From 2017 to 2018, 40 patients with metastatic PAC were enrolled, and tumor tissue and blood samples were collected from 40 and 35 patients, respectively. CtDNA was sequenced by next-generation sequencing (NGS) with a 425-gene capture panel. The association of clinical characteristics, laboratory indicators, and dynamic ctDNA with patient outcomes was analyzed.

**Results:**

Mutations in *KRAS* (87.5%, N = 35) and *TP53* (77.5%, N = 31) were most common in 40 tumor tissue. Patients’ ECOG score, CA19-9, CEA, neutrophil-lymphocyte ratio (NLR), platelet- lymphocyte ratio (PLR) levels and mutations in ≥ 3 driver genes were strongly correlated with patients’ overall survival (OS). Patients’ gender, ECOG score, CA19-9, and CEA levels were associated with progression-free survival (PFS) (P<0.05). In 35 blood samples, univariate analysis showed a significant association between ECOG score, CA19-9, *KRAS* or *CDKN2A* mutation in ctDNA and OS and between CA19-9, *CDKN2A* or *SMAD4* mutation in ctDNA and PFS. Cox hazard proportion model showed that patients’ *CDKN2A* mutation in ctDNA (HR=16.1, 95% CI=4.4-59.1, P<0.001), ECOG score (HR=6.2, 95% CI=2.4-15.7, P<0.001) and tumor location (HR=0.4, 95% CI=0.1-0.9, P=0.027) were significantly associated with OS. Patients’ *CDKN2A* mutation in ctDNA (HR=6.8, 95% CI=2.3-19.9, P=0.001), *SMAD4* mutation in ctDNA (HR=3.0, 95% CI=1.1-7.9, P=0.031) and metastatic organ (HR=0.4, 95% CI=0.2-1.0, P=0.046) were significantly associated with PFS. Longitudinal changes in gene mutation allelic frequency (MAF) value were evaluated in 24 patients. Detection of progression disease (PD) by ctDNA was 0.9 months earlier than by radiological imaging (mean PFS: 4.6m vs 5.5m, P=0.004, paired t-test).

**Conclusions:**

The ctDNA has the potential as a specific survival predictive marker for metastatic PAC patients. Longitudinal ctDNA tracking could potentially help identify disease progression and be a valuable complement for routine clinical markers and imaging.

## Introduction

Pancreatic adenocarcinoma (PAC) is one of the most lethal cancers, having a dismal prognosis. Patients with metastatic pancreatic cancer have a 5-year overall survival rate of less than 2% (1), and less than 5% of patients treated with chemotherapy are projected to survive 5 years (2–5). The prognosis remains dismal despite advances in therapy, most notably the advent of novel chemotherapies and surgical methods, all the more so that cancer is typically found in advanced stages. One reason for poor outcomes may stem from the lack of adequate evaluation methods to select an optimal treatment for an individual patient. Patients with advanced pancreatic cancer must be closely examined to assess tumor burden and therapy response. Although serum protein indicators such as carcinoembryonic antigen (CEA) and carbohydrate antigen 19–9 (CA19–9) are commonly examined, they do not reliably predict prognosis (6). A strict yet minimally intrusive biomarker with high sensitivity and specificity for prognosis prediction and treatment selection is urgently needed.

Blood-based biomarkers such as circulating proteins, RNA, and DNA have gained substantial momentum in cancer diagnosis and treatment stratification. Specifically, detection of circulating tumor DNA (ctDNA) in the blood of patients with breast, colorectal, and lung cancer, among others, has demonstrated therapeutic use in diagnosing patient relapses. In the setting of PAC, the prognostic and predictive value of ctDNA as a clinically meaningful biomarker has been inconsistent (7, 8). Additional sources of DNA and RNA in circulation have been revealed recently in exosomes, which are microvesicles. Prior research has demonstrated the value of analyzing the genomic content of exosomes (exoDNA) as a proxy for the mutational landscapes of existing malignancies and early diagnosis. These 40–150 nm lipid bilayer membrane-bound vesicles are thought to preserve nucleic acid material from a nuclease-mediated breakdown in the plasma, allowing native material to survive in a higher-molecular-weight configuration than ctDNA seen at 170 base pairs. This might increase sensitivity and resolution for high-quality DNA material molecular profiling.

Recent research has shown that ctDNA might be used as a biomarker in various malignancies. In cancer patients, the ctDNA levels rise in response to various physiological events, including inflammation, smoking, and infection (9, 10). CtDNA is primarily generated by apoptosis and necrosis, and apoptosis is significantly amplified in tumor masses due to cancer cell proliferation and fast cell turnover. Thus, the cell debris that macrophages typically phagocytose cannot be entirely eliminated but accumulates and is discharged into the systemic circulation (11–13). Recently, ctDNA was proven as an early marker for therapy response and disease progression in patients with metastatic colorectal and breast cancer (14, 15). In pancreatic cancer, ctDNA is considered a valuable biomarker for predicting the likelihood of postoperative recurrence and assessing patient responses to treatment (16, 17). CtDNA levels may also be adequate clinically for detecting recurrences of pancreatic cancer during surgical follow-up (18–20). Although various studies have examined the efficacy of ctDNA as a biomarker with postoperative pancreatic cancer (18, 21), its prognostic value in metastatic PAC remains unknown. This study aimed to determine the efficacy of ctDNA as a biomarker in metastatic PAC patients.

## Materials and methods

### Patients

The Ethics Committee approved the study of the Chinese PLA General Hospital. All patients provided written informed permission. Patients eligible for enrollment were: a) adults no less than 18 years of age; b) histologically or cytologically confirmed pancreatic adenocarcinoma; c) ECOG performance status of 0 or 2 with life expectancy no less than 12 weeks; d) having at least one measurable distant metastatic lesion by computed tomography (CT) as defined in the Response Evaluation Criteria in Solid Tumors (RECIST) version 1.1. Patients were excluded from the enrollment if they: a) disagreed with genetic testing; b) had peritoneal metastasis only.

### Study design

This prospective cohort study of patients with metastatic PAC enrolled at the Chinese PLA General Hospital from January 2017 to December 2018; eligible patients underwent biopsy, followed by chemotherapy. Tumor tissue from biopsy and pretreatment peripheral blood samples collected before the first cycle of chemotherapy as used for mutational profiling. Plasma samples were prepared within 2 hours after blood collection for DNA extraction. The white blood cells from the buffy coat after plasma preparation were also collected from the same patient at baseline and sequenced as normal controls to identify germline mutations and mutations due to clonal hematopoiesis. The mean sequencing coverage depth of the white blood cells was ~300×. Patients were then scheduled to be followed every 2 months with CT scan and blood collections until CT scan results determined the disease (PD) progression. According to the protocols reviewed, the genetic tests were performed in a centralized clinical testing centre (Nanjing Geneseeq Technology Inc., China; Certified by CAP, CLIA and ISO15189). This study was registered (https://clinicaltrials.gov/number, NCT02124317).

### DNA extraction and targeted next-generation sequencing

Following the manufacturer’s instructions, tissue samples after proteinase K digestion and blood samples were collected into EDTA tubes. And the plasma was separated by centrifugation at 3000 × g for 10 min. QIAmp Circulating Nucleic Acid Kit (Qiagen) was used to extract genomic DNA from plasma and tissue. Tumor samples were extracted from fresh tumor samples using DNeasy Blood & Tissue Kit (Qiagen, Germany). Genomic DNA from white blood cells was extracted using DNeasy Blood & Tissue Kit (Qiagen, Germany) and used as normal control. A260/280 and A260/A230 ratios of purified genomic DNA have been determined by Nanodrop2000 (Thermo Fisher Scientific). Using the dsDNA HS Assay Kit (Life Technologies), all DNA samples were quantified by Qubit 3.0. KAPA Hyper Prep kit (KAPA Biosystems) was used to prepare sequencing libraries with an optimized manufacturer’s protocol. The strands of genomic DNA were sheared into 350-bp fragments using the Covaris M220 (Covaris). Fragments were A-tailored and then ligated sequentially with indexed sequencing adapters, followed by size sorting with Agencourt AMPure XP beads (Beckman Coulter). To maximize ctDNA retrieval from plasma samples, up to 50ng of ctDNA were purified with Agencourt AMPure XP beads (Beckman Coulter) and end-repairing, A-tailing, ligating with customized adapters containing unique molecular indices (UMI), and PCR amplification with primers containing demultiplexing indices sequentially. Agencourt AMPure XP beads were used to purify the PCR-amped libraries. Up to 2 g of total library input was pooled together from different libraries with unique indexes. Human cot-1 DNA (Life Technologies) and xGen Universal blocking oligos (Integrated DNA Technologies) were added as blocking reagents. XGen Lockdown hybridization and wash kit (Integrated DNA Technologies) and Dynabeads M-270 (Life Technologies) were used for the capture reaction. Captured libraries were on-beads PCR amplified with Illumina p5 (5′ AAT GAT ACG GCG ACC GA 3′) and p7 primers (5′ CAA GCA GAA GAC GGC ATA CGA GAT 3′) in KAPA HiFi HotStart ReadyMix (KAPA Biosystems), followed by purification using Agencourt AMPure XP beads. KAPA Library Quantification kit (KAPA Biosystems) was used to quantify libraries. Bioanalyzer 2100 (Agilent Technologies) determined the library fragment size. The target-enriched library was sequenced on the HiSeq4000 NGS platform (Illumina), following the manufacturer’s instructions. A complete list of the genes included in the NGS panel is in **Supplementary Table 1**.

### Data processing and bioinformatics analysis

We used Trimmomatic for FASTQ file quality control (QC) and leading/trailing low quality (quality reading below 30). The remaining reads were mapped to the reference sequence data (Human Genome version 19) using Burrows-Wheeler Aligner (BWA-mem, v0.7.12). Indel realignment and base quality score recalibration were performed with Genome Analysis Toolkit (GATK 3.4.0). Somatic mutations were detected with VarScan2. With default parameters, copy number variations (CNVs) were detected using ADTEx (http://adtex.sourceforge.net). ABSOLUTE estimated tumor purity. Purity-adjusted gene-level and segment-level copy numbers were calculated by CNVKit ADTEx (http://adtex.sourceforge.net). Previously published papers can be used as references (22).

### Statistical analysis

Chi-squared tests were used to compare categorical data (such as gender) between the DNA mutation-positive and- negative groups. Mann–Whitney U tests were used to compare continuous variables (such as CA19-9). Additionally, we examined the associations between ctDNA and CT results and essential blood indicators, such as CA19-9 and CEA. The response rate was assessed using the RECIST version 1.1 criteria. The log-rank test determined correlations between clinical outcomes and ctDNA markers. From the date of the diagnosis until the date of death from any cause, overall survival (OS) was computed. Progression-free survival (PFS) was estimated from the date of the diagnosis to the date of illness recurrence or progression, or death from any cause. The Cox hazard regression approach was utilized to find independent risk variables for OS and PFS. P values of 0.05 or less with two-tailed tests were deemed statistically significant for all statistical analyses. SPSS 25.0 version was used for all statistical studies.

## Result

### Patient characteristics and laboratory indicators

**Figure 1:**
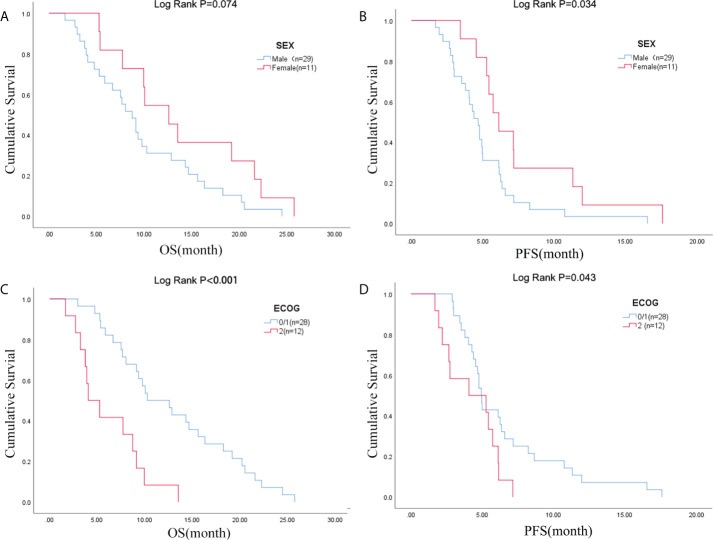
Sex was associated with **(A)** OS and **(B)** PFS; ECOG PS was associated with **(C)** OS and **(D)** PFS. OS, overall survival; PFS, progression-free survival; ECOG PS, Eastern Cooperative Oncology Group Performance Status.

**Figure 2:**
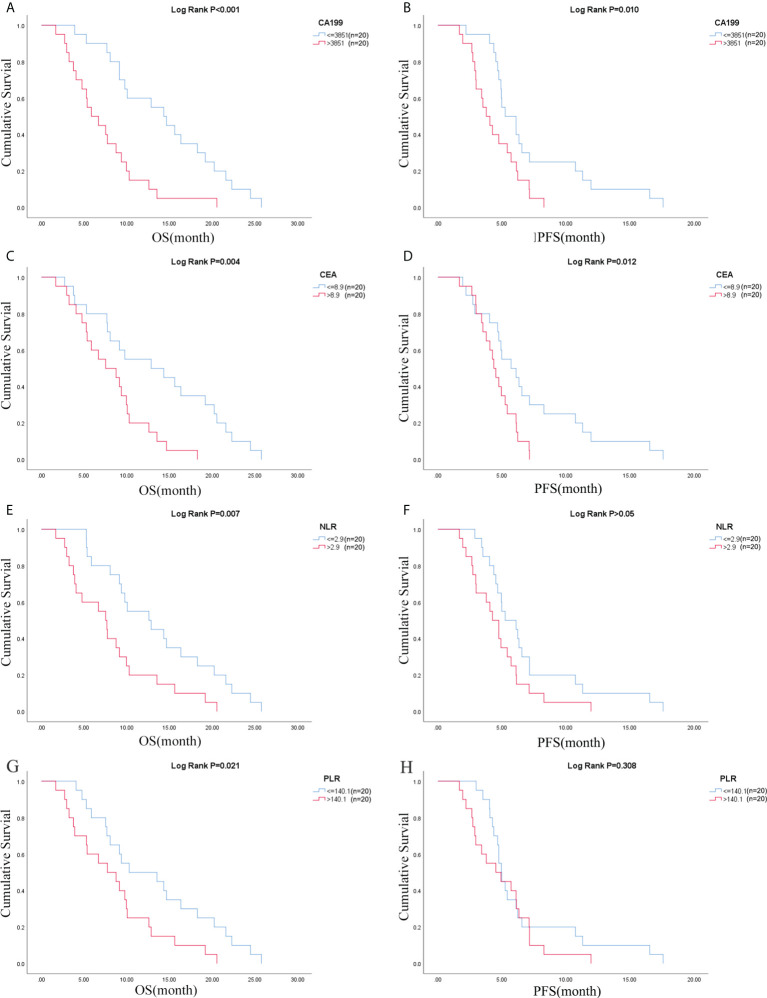
CA19-9 was associated with **(A)** OS and **(B)** PFS; CEA was associated with **(C)** OS and **(D)** PFS; NLR was associated with **(E)** OS and **(F)** PFS; PLR was associated with **(G)** OS and **(H)** PFS. OS, overall survival; PFS, progression-free survival; NLR, neutrophil-lymphocyte ratio; PLR, platelet- lymphocyte ratio.

Due to the exclusion of two patients with peritoneal metastasis only and four patients with insufficient tumor tissue for testing, a final cohort of 40 patients with metastatic pancreatic cancer was formed. Patient characteristics are shown in **Table 1**. The median age of patients at the time of diagnosis was 60 years (range 34-76 years), of whom 45.0% (18/40 cases) were 60 years or older; 72.5% (29/40 cases) were male; 70.0% (28/40 cases) had an ECOG score of 0-1; 32.5% (13/40 cases) had primary pancreatic tumors in the head and neck of the pancreas; 57.5% (23/40 cases) had low-grade tumors; 45.0% (18/40 cases) had only 1 metastatic organ; 85.0% (34/40) had liver metastases; 92.5% were treated with S1 plus nab-paclitaxel which had been evaluated in our phase II trials published before (23). As shown in **Figures 1A, B**, there was a trend towards females (n = 11) having better PFS than males (n = 29) (6.1 versus 4.7 months; p = 0.029) and also better OS, although not statistically significant (12.6 versus 8.7 months; p = 0.074). ECOG was related with better OS (10.3 versus 4.1 months; p <0.001), and better PFS (4.9 versus 4.1 months; p = 0.043) (**Figures 1C, D**; **Table S2**). Peripheral blood laboratory indicators [CA19-9, CEA, neutrophil-lymphocyte ratio (NLR) and platelet- lymphocyte ratio (PLR)] were collected in all patients before the first cycle of chemotherapy. As shown in **Supplementary Table 2**, comparing clinical and laboratory factors with OS and PFS confirmed that patients’ peripheral blood CA19-9, CEA, NLR, and PLR were significantly associated with OS (*P*<0.05). Patients with low CA19-9, low CEA, low NLR, and low PLR had better OS (**Figures 2A, C, E, G**). Patients with low CA19-9 and CEA had better PFS (**Figures 2B, D**). But no significant relation was found between NLR/PLR and PFS (**Figures 2F, H**). Patient’s peripheral blood CA19-9 remarkably correlated with metastatic organ (p = 0.049), while CEA, NLR and PLR levels correlated with patients’ ECOG scores (P<0.05, **Supplementary Table 3**).

**Table 1:** Baseline characteristics of 40 metastatic PAC patients.

Variable	Category	n%
**Age**	<60	22 (55.0%)
	≥60	18 (45.0%)
**Sex**	Male	29 (72.5%)
	Female	11 (27.5%)
**ECOG PS**	0-1	28 (70.0%)
	2	12 (30.0%)
**Primary tumor Location**	Head or Neck	13 (32.5%)
	Body or Tail	27 (67.5%)
**Grade**	Low	23 (57.5%)
	Middle or High	17 (42.5%)
**Metastatic organ**	One	18 (45.0%)
	Multiple	22 (55.0%)
**Metastatic lesion location**	Hepatic metastasis	34 (85.0%)
	Non-hepatic metastasis	6 (15.0%)
**1st line chemotherapy**	S1+nab-paclitaxel	37 (92.5%)
	Germcitabine+nab-paclitaxel	3 (7.5%)

PAC, pancreatic adenocarcinoma; ECOG PS, Eastern Cooperative Oncology Group Performance Status.

### Mutations profile of tumor tissues and pretreatment ctDNA

**Figure 3:**
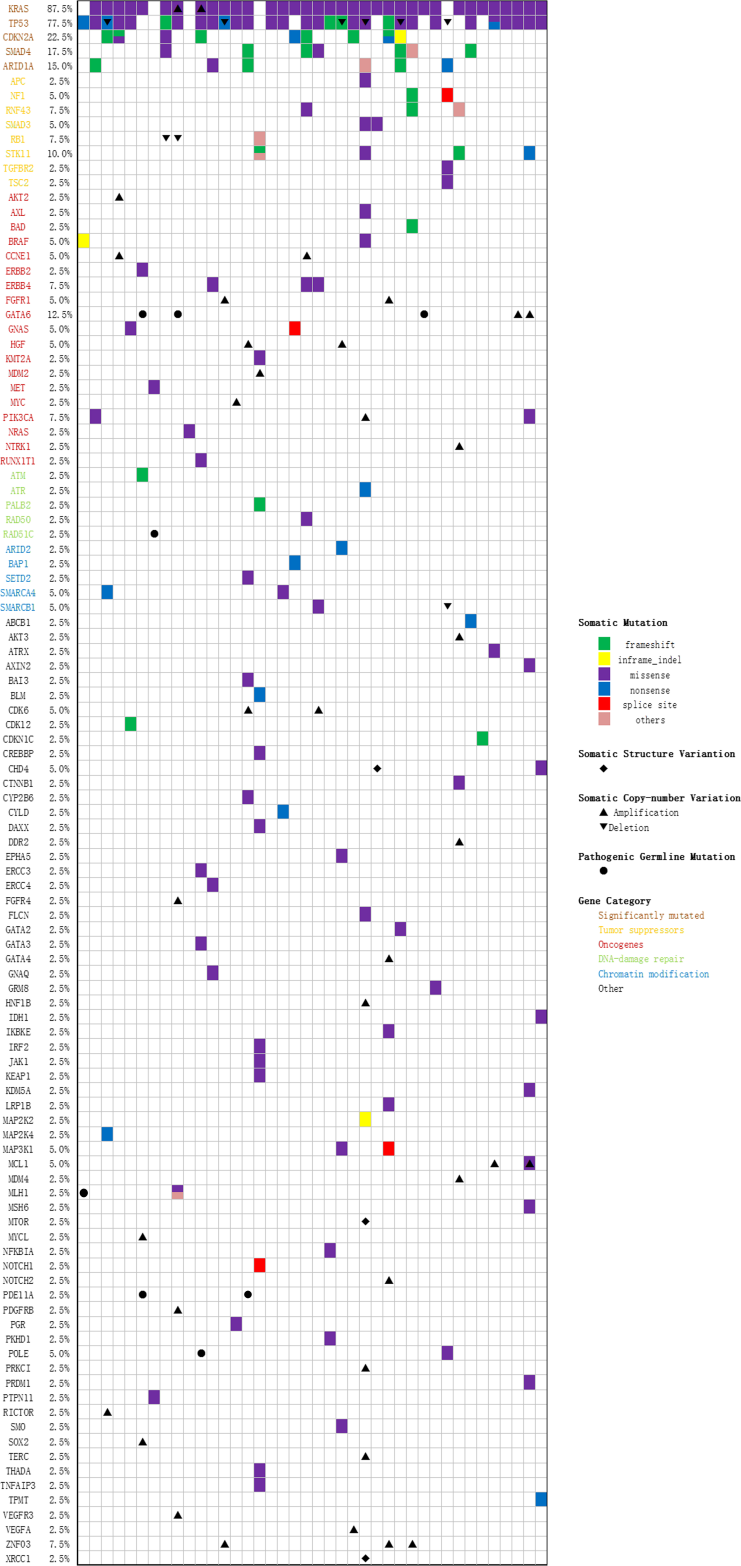
Oncoprint of 425 genes in 40 PAC patients.

40 tumor tissue samples and 35 pretreatment peripheral blood samples (five patients did not have pretreatment plasma sample) were prepared and analyzed by targeted NGS using a pre-designed tracking panel of 425 genes to a mean coverage depth of ~850× for tumor tissues and ~30,000× deep sequencing for ctDNA samples. Genomic DNA from the white blood cells of the buffy coat after plasma separation was also analyzed as the normal control sample for germline variants and clonal hematopoiesis mutation filtering. Of the 40 patients, mutations in *KRAS* and *TP53* were the most frequently detected (87.5% [35/40] and 75% [30/40] patients, respectively). *CDKN2A* and *SMAD4* were detected in 9 (22.5%) and 7 (17.5%) cases, respectively. The results of sequencing of somatic mutations, germline mutations, and CNV (copy number variation) in the patients are shown in **Supplementary Table 4** and **Figure 3**. The mean number of somatic mutations in the patients was 4.6; germline mutations were present in 7 patients, and CNV was present in 23 patients. Concordant alterations in plasma ctDNA and tumor tissue DNA was confirmed in 35 patients (**Supplementary Table 5**). In univariate analysis, four primary driver genes were not a predictor for OS or PFS on their own. However, after combination, we found that patients with mutations in ≥ 3 driver genes had worse OS than ≤ 2 mutated genes (6.7 versus 10.1 months, p = 0.048, **Supplementary Table 2**).

### ctDNA as a prognostic marker in advanced pancreatic cancer

**Figure 4:**
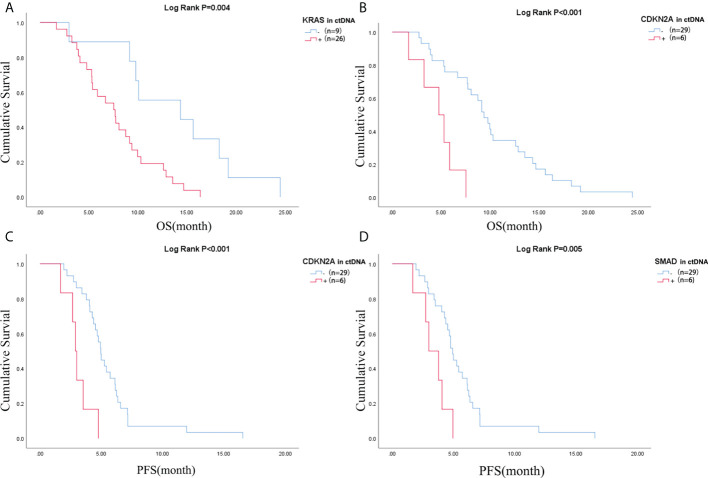
Difference of OS of patients in **(A)** *KRAS* mutation negative and positive in ctDNA group and **(B)** *CDKN2A* mutation negative and positive in ctDNA group; Difference of PFS of patients in **(C)** *CDKN2A* mutation negative and positive in ctDNA group and **(D)** *SMAD4* mutation negative and positive in ctDNA group. OS, overall survival; PFS, progression-free survival; ctDNA, circulating tumor DNA.

Pretreatment blood samples from 35 of 40 patients were further analyzed to determine the underlying clinicopathological features of ctDNA shedding in PAC. Of the 35 pretreatment peripheral blood samples, mutations in *KRAS*, *TP53*, *CDKN2A* and *SMAD4* were detected in 26 (74.3% [26/35]), 24 (68.6% [24/35]), 6 (17.1% [6/35]) and 6 (17.1% [6/35]) cases, respectively. The results showed that positive *KRAS* mutation in ctDNA was significantly correlated with ECOG score (P=0.015), while positive *TP53* mutation in ctDNA was significantly correlated with metastatic lesion location (P=0.026) (**Supplementary Table 6**). We performed a Kaplan-Meier analysis on pretreatment blood samples from 35 patients to assess the predictive value of ctDNA mutations (**Table 2**). By logarithmic test, we found a significant association between the presence of *KRAS* ctDNA and OS (*KRAS* mutation-positive vs negative: 7.5m vs 14.3m, p=0.004, **Figure 4A**), also between the *CDKN2A* ctDNA and OS (*CDKN2A* mutation-positive vs negative: 4.8m vs 9.4m, p<0.001, **Figure 4B**). We also found a significant association between the presence of *CDKN2A* ctDNA and PFS (*CDKN2A* mutation-positive vs negative: 2.9m vs 5.0m, p<0.001, **Figure 4C**), also between the *SMAD4* ctDNA and PFS (*SMAD4* mutation-positive vs negative: 3.0m vs 4.9m, p=0.005, **Figure 4D**). In addition, we performed Cox regression analysis, summarized in **Table 3**. Cox hazard proportion model showed that patients’ *CDKN2A* mutation in ctDNA (HR=16.1, 95% CI=4.4-59.1, P<0.001), ECOG score (HR=6.2, 95% CI=2.4-15.7, P<0.001) and primary tumor location (HR=0.4, 95% CI=0.1-0.9, P=0.027) were significantly associated with OS. Patients’ *CDKN2A* mutation in ctDNA (HR=6.8, 95% CI=2.3-19.9, P=0.001), *SMAD4* mutation in ctDNA (HR=3.0, 95% CI=1.1-7.9, P=0.031) and metastatic organ (HR=0.4, 95% CI=0.2-1.0, P=0.046) were significantly associated with PFS.

**Table 2:** Association analysis of four main driver genes mutation in ctDNA with survival in 35 metastatic PAC patients.

Variables	No.	OS				PFS		
		OS	95%CI	*p*		PFS	95%CI	*p*
*KRAS* in ctDNA				0.004				0.167
*KRAS* -	9	14.3	1.8-26.8			4.9	4.2-5.7	
*KRAS* +	26	7.5	5.2-9.9			4.3	3.4-5.2	
*TP53* in ctDNA				0.374				0.579
*TP53* -	11	9.8	7.3-12.3			4.5	3.9-5.2	
*TP53* +	24	7.7	5.3-10.0			4.8	3.9-5.6	
*CDKN2A* in ctDNA				<0.001				<0.001
*CDKN2A* -	29	9.4	8.0-10.7			5.0	4.6-5.4	
*CDKN2A* +	6	4.8	2.3-7.2			2.9	2.5-3.3	
*SMAD4* in ctDNA				0.083				0.005
*SMAD4* -	29	9.1	8.0-10.2			4.9	4.5-5.4	
*SMAD4* +	6	4.1	0-8.8			3.0	1.7-4.3	

OS, overall survival; PFS, progression-free survival; PAC, pancreatic adenocarcinoma; CI, confidence interval; ctDNA, circulating tumor DNA.

**Table 3:** Cox proportional hazard analysis of clinical variable and ctDNA for the prediction of OS and PFS in 35 metastatic PAC.

Variables	OS		Variables		PFS		
	HR (95%CI)	*P*			HR (95%CI)		*P*
*CDKN2A* in ctDNA	16.1 (4.4-59.1)	<0.001		*CDKN2A* in ctDNA	6.8 (2.3-19.9)	0.001	
ECOG PS	6.2 (2.4-15.7)	<0.001		*SMAD4* in ctDNA	3.0 (1.1-7.9)	0.031	
Primary tumor location	0.4 (0.1-0.9)	0.027		Metastatic organ	0.4 (0.2-1.0)	0.046	

Adjusted for: Age, Sex, ECOG PS, Primary tumor location, Grade, Metastatic organ, Metastatic lesion location, CA19-9, CEA, NLR, PLR and KRAS/TP53/CDKN2A/SMAD4 in ctDNA; ctDNA, circulating tumor DNA; OS, overall survival; PFS, progression-free survival; CI, confidence interval; PAC, pancreatic adenocarcinoma; ECOG PS, Eastern Cooperative Oncology Group Performance Status.

### Longitudinal ctDNA analysis for disease monitoring

**Figure 5:**
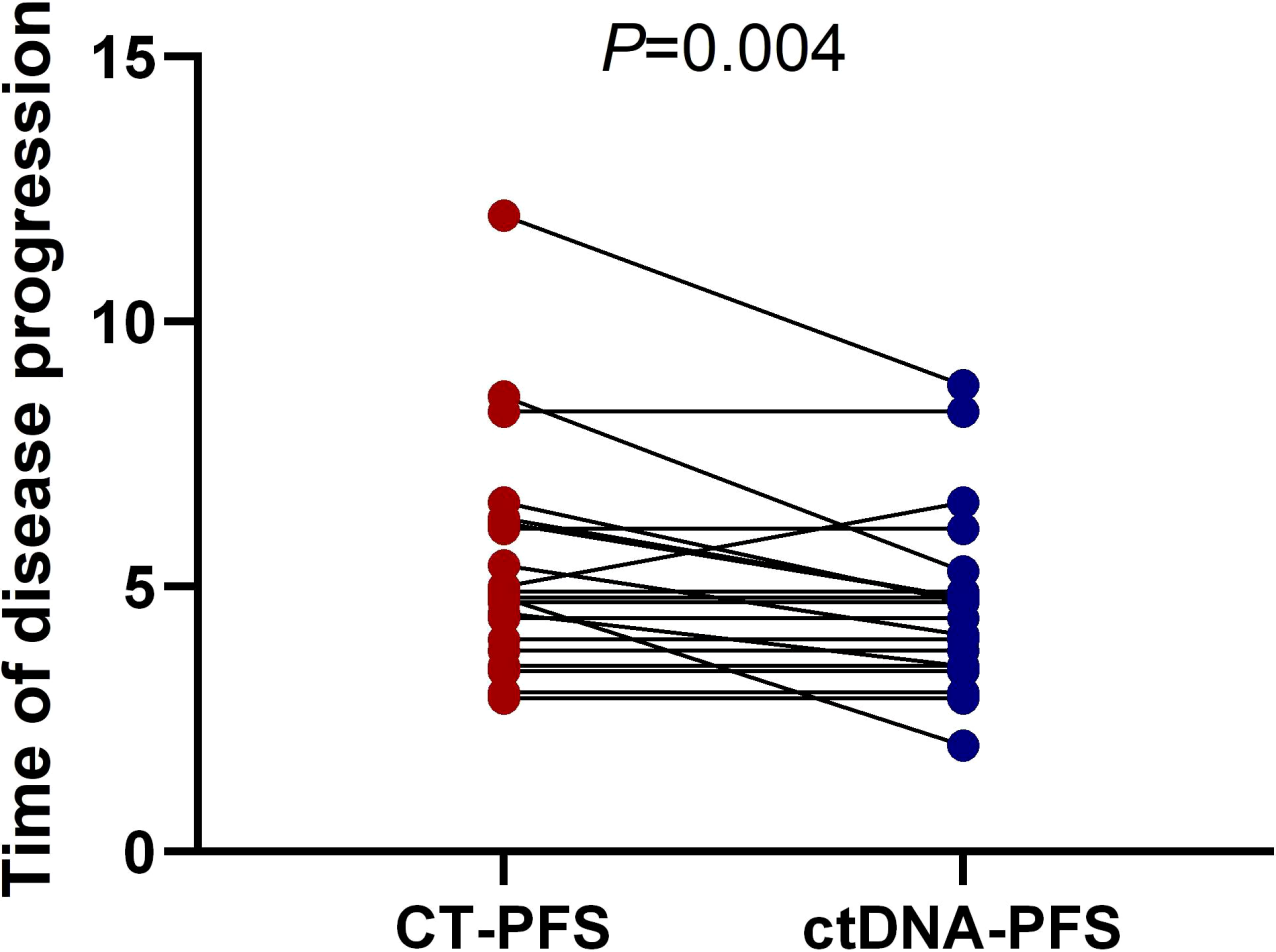
Longitudinal ctDNA analysis for disease progression monitoring. The comparison of the disease progression time measured by ctDNA versus CT (4.6month vs 5.5month, p = 0.004, two-sided Wilcoxon two-sample paired signed-rank test, p = 0.004). CT, computed tomography; PFS, progression-free survival; ctDNA, circulating tumor DNA.

**Figure 6:**
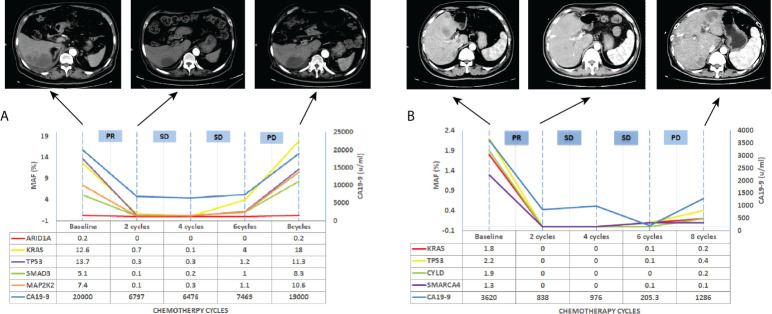
Longitudinal ctDNA analysis, CA19-9 value and CT scan for disease progression monitoring. **(A)** CT scan, CA19-9 value and MAF of ctDNA detection for patients A, **(B)** CT scan, CA19-9 value and MAF of ctDNA detection for patients B. MAF, mutation allelic frequency; PR, partial response; SD, stable disease; PD, progressive disease.

We further investigated whether longitudinal ctDNA mutation allelic frequency (MAF) tracking during chemotherapy can serve as a dynamic biomarker for disease progression monitoring. Of the 35 patients with the pretreatment blood sample, 24 patients underwent at least two peripheral blood sampling during chemotherapy (every 2 cycles) until a CT scan determined progression disease (PD). After comparative analysis, the results showed that ctDNA-positive PFS was earlier than the radiological PFS in 11 patients but was inconsistent in 1 patient (a more than 2-fold increase in MAF values for any of four primary driven genes compared with the previous test result or a change from negative to positive in any gene was considered to indicate ctDNA-positive disease progression). The other patients’ ctDNA changes were consistent with the patient’s tumor imaging presentation, and the corresponding individual data were referred to the **Supplemental Figures 1**–**3**. The mean time of ctDNA-positive PFS was 0.9 months lower than radiological PFS (4.6m vs 5.5m, p=0.004, paired t-test, **Figure 5**). For example, patient A was considered a radiological disease progression after 8 cycles of chemotherapy. However, ctDNA analysis had already shown a notable increase in MAF of multiple genes after 6 cycles of chemotherapy, while the CA19-9 value remained stable (**Figure 6A**). The ctDNA of patient B was reduced to negative after 4 cycles of chemotherapy. Interestingly, the ctDNA analysis of the *KRAS* and *TP53* gene turn to positive after 6 cycles of chemotherapy, while the CA19-9 value was continuously dropping, and the tumor was stable on CT (**Figure 6B**). These results suggest that longitudinal ctDNA tracking could potentially help identify disease progression and be a helpful complement for routine clinical markers and imaging.

## Discussion

Conducting a thorough molecular analysis of tissue biopsy samples is typically one of the first stages toward establishing a cancer diagnosis. However, the therapeutic value of this technique is frequently compromised in patients with pancreatic cancer due to the difficulties of acquiring appropriate tissue samples (24). As a result, much effort has been directed to developing and implementing highly accurate and less intrusive blood tests for cancer screening. Although various serological biomarkers, such as CEA and CA 19–9, are employed in clinical practice, they are frequently insufficiently sensitive or specific for prognosis in patients with pancreatic cancer (25). Recent studies have demonstrated that ctDNA—which can be obtained *via* liquid biopsies and detected with high sensitivity *via* next-generation sequencing or ddPCR—is released from the primary tumor and/or its metastases (26–29) and thus could be used to detect cancer and/or its recurrence at very early stages. While some studies have demonstrated the clinical value of ctDNA in patients with pancreatic cancer, few have done so with sufficiently high sample numbers. The purpose of this study was to determine the clinical value of ctDNA as a predictive biomarker in patients with metastatic pancreatic cancer.

*KRAS*, *TP53*, *CDKN2A*, and *SMAD4* genes are the most commonly mutated genes in the current investigation. This result is consistent with the database of COSMIC (30). Whole-genome and exome sequencing has been used to study the molecular genetic landscape of pancreatic cancer (31–35). The oncogene *KRAS* is present in greater than 90% of PanIN-1 (Pancreatic intraepithelial neoplasia, PanIN) lesions. Constitutive *KRAS* activation leads to the activation of several complex downstream pathways, such as the MAPK (Mitogen-Activated Protein Kinase) pathway and PI3K pathway. The protein encoded by *TP53* (p53) gene plays an important role in DNA repair mechanisms, cell growth arrest and activation of apoptosis following the cellular injury. *TP53* is targeted late in PanIN progression, usually not until PanIN-3. The *CDKN2A* gene (chromosome 9p) is the most commonly inactivated tumor suppressor gene, targeted in 95% of ductal adenocarcinomas. The protein product of the *CDKN2A* gene, p16^INK4A^, normally functions to inhibit the G1 phase of the cell cycle by inhibiting the cyclin D-dependent kinases (CDK4 and CDK6) and, therefore, the phosphorylation of retinoblastoma protein. Loss of p16^INK4A^ occurs in an early stage of pancreatic carcinogenesis, causing faster progression of Pan-IN lesions to an invasive tumor (36). *SMAD4* gene (chromosome 18q21.2) codes for the protein Smad4, transforming growth factor β (TGF-β) signalling. It is considered that the inactivation of *SMAD4* is remarkably associated with the invasiveness of cancer. The progression of pancreatic carcinogenesis arises from low‐grade dysplasia carrying *KRAS* mutation to high‐grade invasive carcinomas when *TP53* mutations have occurred, with intermediate stages involving inactivation of *CDKN2A* and *SMAD4*. Emerging strategies are being developed to target these genes. Advances have been achieved in clinical and preclinical trials of therapies, and further investigations are warranted (37). We detected *KRAS* and *TP53* mutated tumor tissue in 87.5% and 75% of the 40 patients individually. This positivity rate is consistent with that described in other studies (70-90%).

Although the correlation between the *KRAS* gene in tumor tissue and survival was controversial in PAC, A combination of *KRAS* and *TP53* gene was reported to be an independent predictor of clinical outcome (38). The prognostic and therapeutic roles of *CDKN2A* gene mutation have not been extensively investigated yet. Research (39) suggested that the *CDKN2A* functional inactivation caused by modifications and deep deletions predicts poor prognosis in PAC patients. Besides, *CDKN2A* inactivation results in the upregulation of estrogen response-related genes, which can be reversed by paclitaxel. Mutations in *SMAD4* are detected in 15-30% of patients with PAC (40). It was also found to be an independent prognostic factor in PAC patients (41). All those results suggested that these gene mutations might not provide sufficient prognostic discrimination in PAC on their own. But after combing multiple genes, several groups (42, 43) revealed that it is remarkably related to worse survival. Our trial did not show any significant association between four primary driver genes in tumor tissue with prognosis. However, patients with mutations in ≥ 3 driver genes had worse OS than ≤ 2 mutated genes (6.7 versus 10.1 months, p = 0.048). Similar conclusion had been drawn in other research (38). It may be of value in stratifying patients for different treatment regimens.

Due to the complex genomic landscape with frequent copy number changes and point mutations, and the randomness and uncertainty of gene changes during the disease progression, ctDNA is still a controversial prognostic biomarker in pancreatic cancer (42, 43). In addition, incongruent testing methods in research may result in different conclusions. Nevertheless, several researchers (8, 44, 45) demonstrated that mutation in ctDNA is a more useful factor than mutation in tissue DNA in predicting prognosis. These results may reflect an actual pathological condition because ctDNA is believed to be produced from the potent cells concerning invasiveness and a rapid life-to-death cycle. Also, ctDNA may make it possible to conquer the limitation of tumor heterogeneity. Although ctDNA has shown a better representation of heterogeneity, data have revealed tissue-derived DNA and ctDNA are comparable with high concordance rates (38, 46, 47). Our data have reached the same conclusion. A recently published article revealed that *KRAS* and *TP53* alterations in ctDNA were associated with both PFS and OS, it also demonstrated that patients who harbored multiple somatic alterations have a worse OS (38). Mutations in *SMAD4* are related to advanced disease, poor OS and recurrence in resectable pancreatic cancer (41). Our trial showed that *KRAS* and *CDKN2A* mutations in ctDNA were significantly associated with OS, while *CDKN2A* and *SMAD4* mutations in ctDNA were correlated with PFS. Cox analysis concluded that *CDKN2A* mutation in ctDNA was independent prognostic factors for OS, and *CDKN2A* or *SMAD4* mutation in ctDNA were independent prognostic factors for PFS. The current study is the first to report *CDKN2A* and *SMAD4* mutations in ctDNA as a prognostic factor for advanced Chinese PAC in NGS. Therefore, our observation, combined by findings from other groups, corroborates the hypothesis that ctDNA has the potential as a specific survival predictive marker for pancreatic cancer and that ctDNA could potentially be used as an indicator for selecting appropriate treatment regimens (17, 38). We may choose FOLFIRINOX (fluorouracil, folinic acid [leucovorin], irinotecan, and oxaliplatin) and gemcitabine plus nanoparticle albumin-bound paclitaxel (nab-paclitaxel) as prior treatment regimens for patients who have one or more main driver genes mutation in ctDNA testing.

Conventional imaging techniques based on optical measurements like CT only reflect the relation between the optical measurement and the actual tumor size. It is nevertheless limited due to deviations produced by technological limitations in the use of algorithms and sampling methods, discordance between the visual and exact active tumor sizes, and tumor necrosis (48). In many situations, CT or MRI cannot provide additional information regarding the basic biological properties of some malignancies, and ctDNA may be more suitable for distinguishing between necrosis and disease relapse and predicting the prognosis of cancer. Although CA19-9 has been proven to be the most specific tumor marker in PAC, about 15% of PAC patients are CA19-9 non-secretors due to the Lewis antigen of red cell phenotyping, which is indispensable for expressing the CA19-9 antigen. The limitation of CA19-9, such as its long half-life and poor sensitivity, limit its role in the real-time observation of efficacy. The ctDNA is a product of tumor tissue metabolism and is thought to reflect the tumor load in patients to some extent. A group of researchers (47) described the relationship between ctDNA concentrations and tumor load. They found that the concentration of ctDNA is positively correlated with tumor load and can be used to monitor the efficacy of tumor therapy. They also indicated that the change in ctDNA concentration was consistent with the imaging assessment and a more sensitive efficacy assessment marker than plasma CA19-9. Tjensvoll et al. (49) compared ctDNA changes during first-line chemotherapy with imaging and CA19-9 levels in nine patients with advanced pancreatic cancer. They found that the trend of ctDNA changes corresponded with CA19-9 changes and tumor efficacy assessment according to RECIST criteria, with one patient’s ctDNA indicating tumor progression one month earlier than the imaging method and another patient two months earlier. Kruger et al. (8) reached the same conclusion and proved that for ^mut^ *KRAS* ctDNA, any increase from base-line was considered meaningful. But these studies used *KRAS* mutation as the indication of ctDNA. That means patients with wild-type *KRAS* gene or with new gene mutation developed during therapy would be missed. In another study including 104 patients of advanced pancreatic cancer, the median variant allele frequency (VAF) was 0.45% (rang 0-55%). Researchers demonstrated that VAF changes between treatments and was related to outcomes (38). Tao W et al. (47) described the ctDNA analysis as a way to monitor tumor burden based on a 560 genes panel in 17 advanced PAC. They focused on the four common driver genes and provided evidence that ctDNA level correlates with tumor burden. In our study, a panel of 425 genes was conducted. The mutation allelic frequency (MAF) level change of four primary genes compared with the previous test result or any new gene mutation developed during chemotherapy indicated ctDNA-positive. By longitudinal ctDNA tracking during chemotherapy in 24 patients, we found an earlier occurrence of MAF increase than of disease progression evaluated by CT imagine. And Only 1 patient showed the opposite result. To the best of our knowledge, similar research in metastatic PAC was not published before. In clinical practice, we may deem the increase of MAF in ctDNA an indicator of accelerated tumor cell proliferation, which happened before the tumor volume change displayed on the CT image. An earlier intervention in disease progression may improve patient outcomes to some degree. Further research is needed to demonstrate this idea. Therefore, a ctDNA test may be recommended instead of the traditional blood tumor marker or even CT image, as suggested by others (50, 51).

There were limitations in our study. First, this is a single-arm, single-centre study. Second, although a 425 gene panel was conducted in our research, the CNV and functional pathways relative to oncogenesis were not analyzed due to the small number of patients. Third, most patients included in this research were not treated with the currently most active regiments including mFOLFIRINOX and gemcitabine plus nab-paclitaxel. Fourth, the small sample size was also a limitation. A subsequent study with higher sample size will be carried out to confirm current results and explore more findings.

In conclusion, this study suggests that evaluation of ctDNA is a prognostic tool that may be applicable to clinical practice. A combination evaluation using imaging and genomic assessment can potentially complement therapeutic strategies, and facilitate a more accurate prognosis prediction. In addition, the evaluation of ctDNA may be used as an alternative genotyping assay in cases where tumor samples are scarce or hardly obtainable. Further development of such combination methods may also help devise individualized treatment strategies for PAC.

## Data availability statement

The datasets presented in this study can be found in online repositories. The names of the repository/repositories and accession number(s) can be found in the article/**Supplementary Material**.

## Ethics statement

The studies involving human participants were reviewed and approved by Chinese PLA General Hospital. The patients/participants provided their written informed consent to participate in this study.

## Author contributions

SG, GD and JS contributed equally to this work as writer and collect data. GHD and NQ as co-corresponding author as supervision. QH, YL, TX, LD and TY contributed to analysis. All authors contributed to the article and approved the submitted version.

## Conflict of interest

The authors declare that the research was conducted in the absence of any commercial or financial relationships that could be construed as a potential conflict of interest.

## Publisher’s note

All claims expressed in this article are solely those of the authors and do not necessarily represent those of their affiliated organizations, or those of the publisher, the editors and the reviewers. Any product that may be evaluated in this article, or claim that may be made by its manufacturer, is not guaranteed or endorsed by the publisher.
