# Supplementary material for: Evaluation of circulating tumor DNA as a prognostic biomarker for metastatic pancreatic adenocarcinoma
Source: Front Oncol. 2022 Aug 23;12:926260. doi: 10.3389/fonc.2022.926260 (PMC9446234; doi:10.3389/fonc.2022.926260)
Supplement: Supplementary file 1 [file DataSheet_1.pdf]

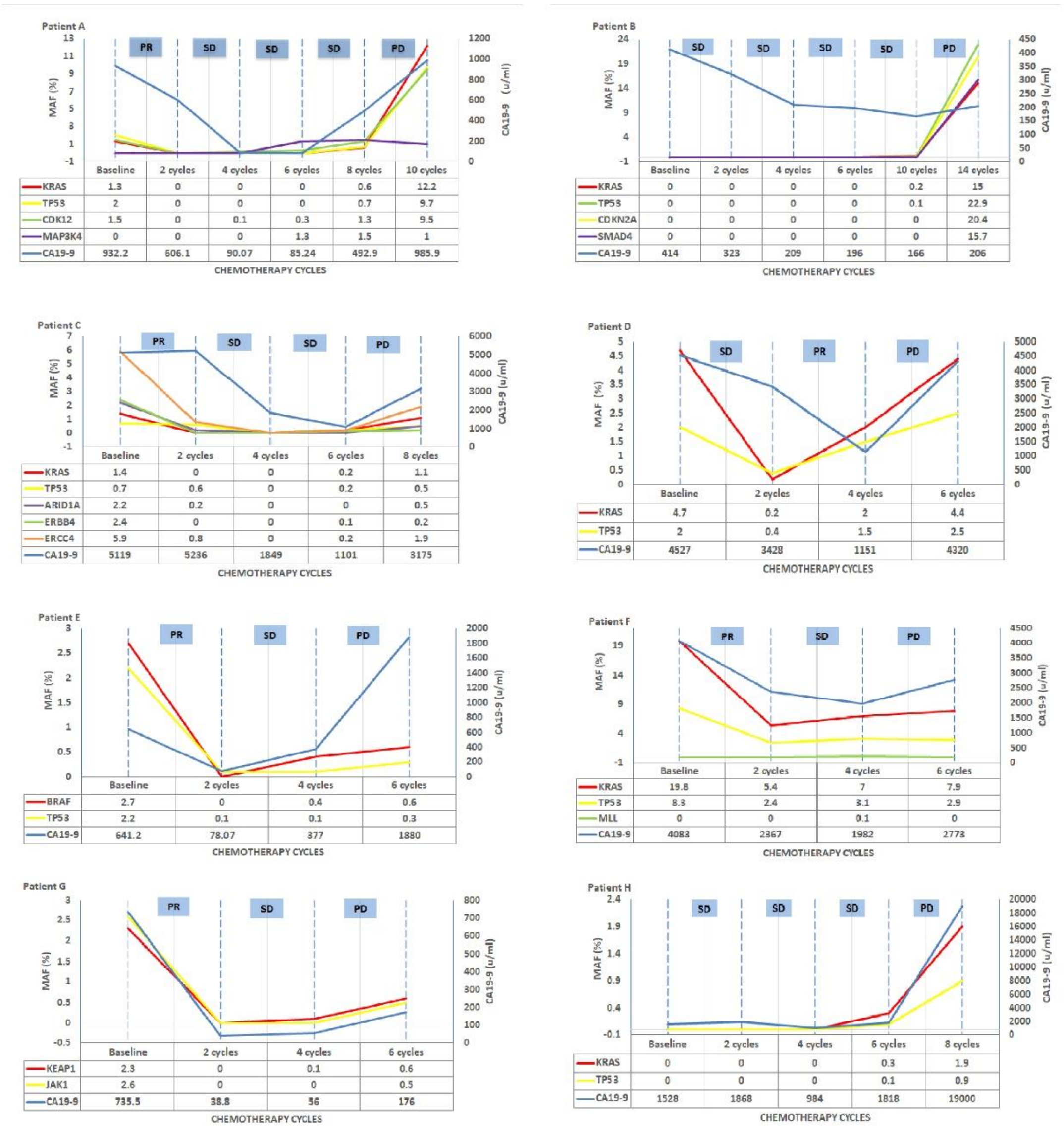


**Supplementary figure 1.** Longitudinal ctDNA analysis and CA19-9 value for disease progression monitoring in 6 metastatic PAC patients （patient A- patient H）. MAF, mutation allelic frequency; PR, partial response; SD, stable disease; PD, progressive disease; PAC, pancreatic adenocarcinoma.

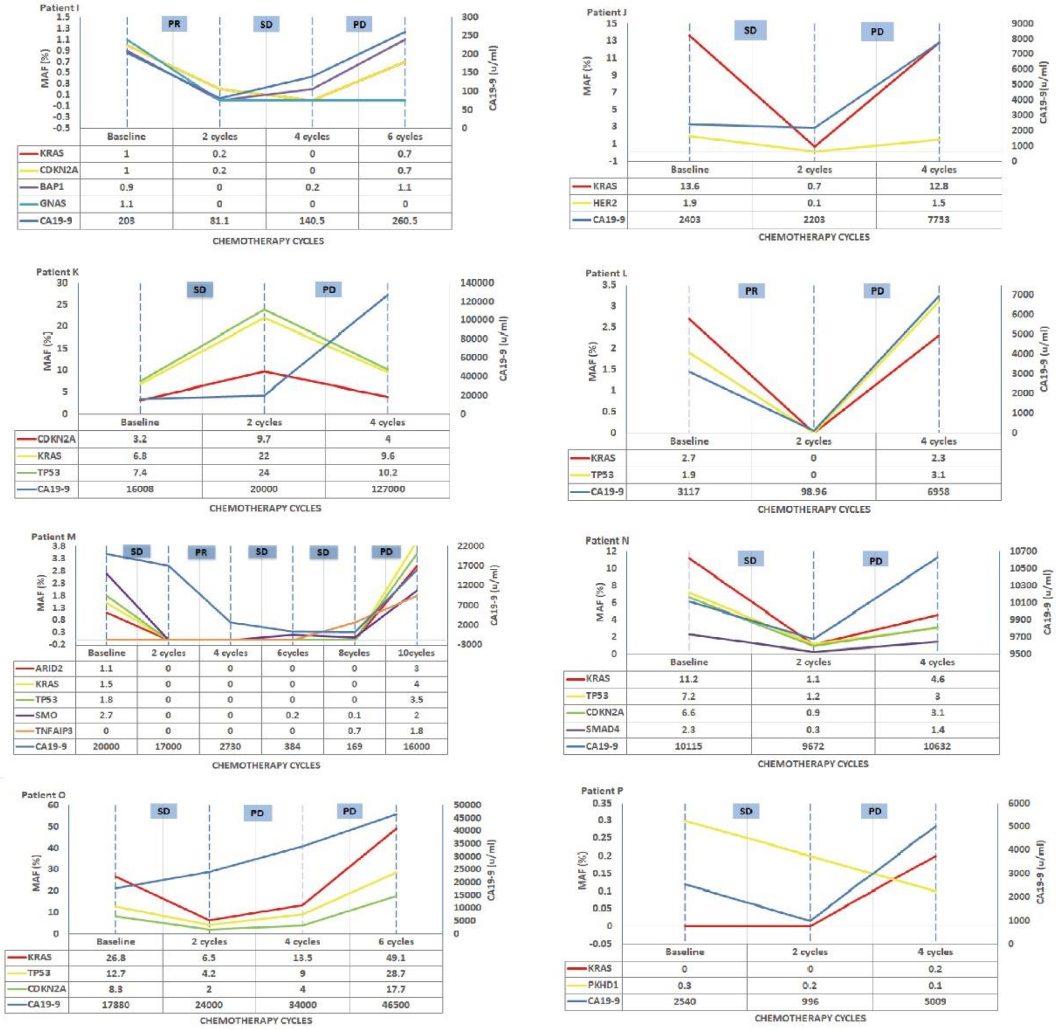


**Supplementary figure 2.** Longitudinal ctDNA analysis and CA19-9 value for disease progression monitoring in 6 metastatic PAC patients （patient I- patient P）. MAF, mutation allelic frequency; PR, partial response; SD, stable disease; PD, progressive disease; PAC, pancreatic adenocarcinoma.

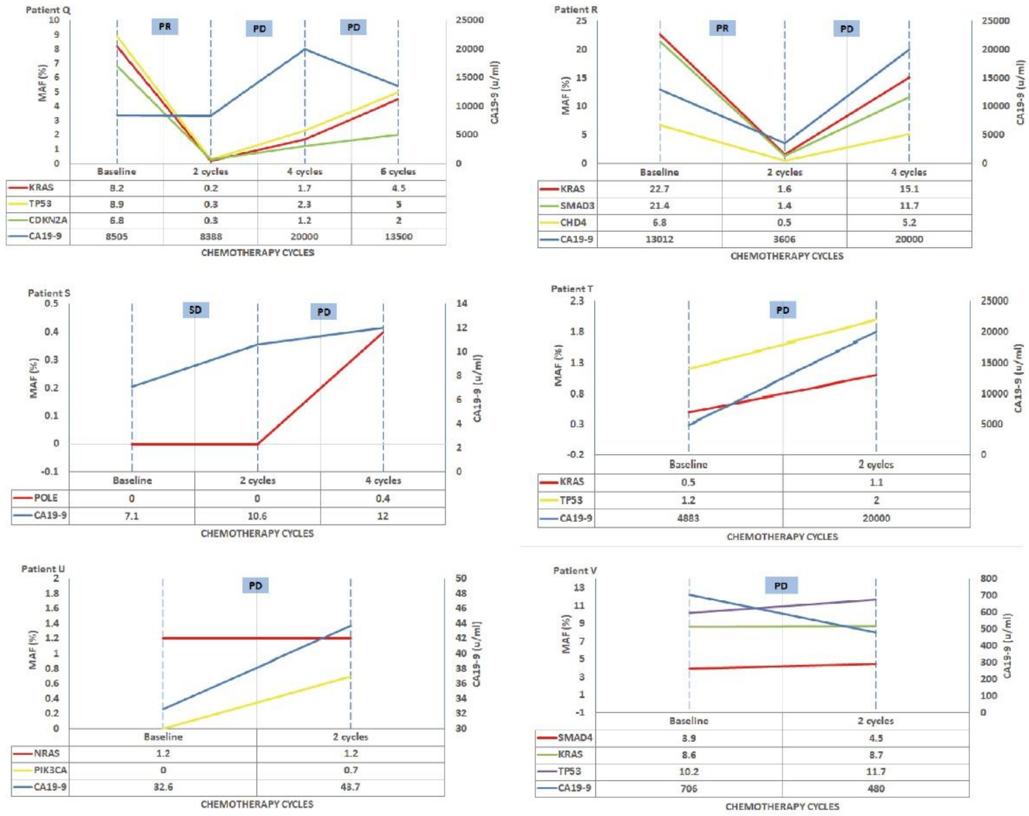


**Supplementary figure 3.** Longitudinal ctDNA analysis and CA19-9 value for disease progression monitoring in 6 metastatic PAC patients （patient Q- patient V）. MAF, mutation allelic frequency; PR, partial response; SD, stable disease; PD, progressive disease; PAC, pancreatic adenocarcinoma.
