# Supplementary material for: Evaluation of circulating tumor DNA as a prognostic biomarker for metastatic pancreatic adenocarcinoma
Source: Front Oncol. 2022 Aug 23;12:926260. doi: 10.3389/fonc.2022.926260 (PMC9446234; doi:10.3389/fonc.2022.926260)
Supplement: Supplementary file 2 [file DataSheet_2.docx]

Supplementary Material

Table S1. List of 425 panel genes testified by NGS.

| *ABCB1* | *BRIP1* | *CYP2D6* | *FBXW7* | *IRF2* | *MRE11A* | *PIK3CA* | *RICTOR* | *TERT* |
| --- | --- | --- | --- | --- | --- | --- | --- | --- |
| *ABCB4* | *BTG2* | *CYP3A4*4* | *FGF19* | *JAK1* | *MSH2* | *PIK3R1* | *RNF43* | *TET2* |
| *ABCC2* | *BTK* | *CYP3A5* | *FGFR1* | *JAK2* | *MSH6* | *PIK3R2* | *ROS1* | *TGFBR2* |
| *ADH1A* | *BUB1B* | *DAXX* | *FGFR2* | *JAK3* | *MTHFR* | *PKHD1* | *RPTOR* | *THADA* |
| *ADH1B* | *c11orf30* | *DDR2* | *FGFR3* | *JARID2* | *MTOR* | *PLAG1* | *RRM1* | *TMEM127* |
| *ADH1C* | *CASP8* | *DENND1A* | *FGFR4* | *JUN* | *MUTYH* | *PLK1* | *RUNX1* | *TMPRSS2* |
| *AIP* | *CBL* | *DHFR* | *FH* | *KDM5A* | *MYC* | *PMS1* | *RUNX1T1* | *TNFAIP3* |
| *AKT1* | *CBLB* | *DICER1* | *FLCN* | *KDM6A* | *MYCL* | *PMS2* | *SBDS* | *TNFRSF11A* |
| *AKT2* | *CCND1* | *DLL3* | *FLT1* | *KDR* | *MYCN* | *POLD1* | *SDC4* | *TNFRSF14* |
| *AKT3* | *CCNE1* | *DNMT3A* | *FLT3* | *KEAP1* | *MYD88* | *POLD3* | *SDHA* | *TNFRSF19* |
| *ALDH2* | *CD274* | *DPYD* | *FLT4* | *KIF1B* | *MYH9* | *POLE* | *SDHB* | *TNFSF11* |
| *ALK* | *CD74* | *DUSP2* | *FOXA1* | *KIF5B* | *NAT1* | *POLH* | *SDHC* | *TOP1* |
| *AMER1* | *CDA* | *EGFR* | *FOXP1* | *KIT* | *NBN* | *POT1* | *SDHD* | *TOP2A* |
| *APC* | *CDC73* | *EML4* | *FRG1* | *KITLG* | *NCOR1* | *PPARD* | *SEPT9* | *TP53* |
| *AR* | *CDH1* | *EP300* | *GATA1* | *KLLN* | *NF1* | *PPP2R1A* | *SETBP1* | *TP63* |
| *ARAF* | *CDK10* | *EPAS1* | *GATA2* | *KMT2A* | *NF2* | *PRDM1* | *SETD2* | *TPMT* |
| *ARID1A* | *CDK12* | *EPCAM* | *GATA3* | *KMT2B* | *NFE2L2* | *PRF1* | *SF3B1* | *TSC1* |
| *ARID1B* | *CDK4* | *EPHA2* | *GATA4* | *KMT2C* | *NFKBIA* | *PRKACA* | *SGK1* | *TSC2* |
| *ARID2* | *CDK6* | *EPHA3* | *GATA6* | *KMT2D* | *NKX2-1* | *PRKACG* | *SLC34A2* | *TSHR* |
| *ARID5B* | *CDK8* | *EPHA5* | *GNA11* | *KRAS* | *NKX2-4* | *PRKAR1A* | *SLC3A2* | *TTF1* |
| *ASCL4* | *CDKN1A* | *EPHB2* | *GNAQ* | *LHCGR* | *NOTCH1* | *PRKCI* | *SLC7A8* | *TUBB3* |
| *ASXL1* | *CDKN1B* | *ERBB2* | *GNAS* | *LMO1* | *NOTCH2* | *PRKDC* | *SMAD2* | *TUBB4A* |
| *ATF1* | *CDKN1C* | *ERBB2IP* | *GRIN2A* | *LRP1B* | *NOTCH3* | *PRSS1* | *SMAD3* | *TUBB4B* |
| *ATIC* | *CDKN2A* | *ERBB3* | *GRM3* | *LYN* | *NPM1* | *PRSS3* | *SMAD4* | *TUBB6* |
| *ATM* | *CDKN2B* | *ERBB4* | *GRM8* | *LZTR1* | *NQO1* | *PTCH1* | *SMAD7* | *TYMS* |
| *ATR* | *CDKN2C* | *ERCC1* | *GSTM1* | *MAP2K1* | *NRAS* | *PTEN* | *SMARCA4* | *U2AF1* |
| *ATRX* | *CEBPA* | *ERCC2* | *GSTM4* | *MAP2K2* | *NRG1* | *PTK2* | *SMARCB1* | *UGT1A1* |
| *AURKA* | *CEP57* | *ERCC3* | *GSTM5* | *MAP2K4* | *NSD1* | *PTPN11* | *SMO* | *VAMP2* |
| *AURKB* | *CHD4* | *ERCC4* | *GSTP1* | *MAP3K1* | *NTRK1* | *PTPN13* | *SOS1* | *VEGFA* |
| *AXIN2* | *CHEK1* | *ERCC5* | *GSTT1* | *MAP3K4* | *NTRK2* | *PTPRD* | *SOX1* | *VHL* |
| *AXL* | *CHEK2* | *ESR1* | *HDAC2* | *MAP4K3* | *NTRK3* | *QKI* | *SOX14* | *WAS* |
| *B2M* | *CREBBP* | *ETV1* | *HDAC9* | *MAX* | *PAK3* | *RAC1* | *SOX2* | *WISP3* |
| *BAD* | *CRKL* | *ETV4* | *HGF* | *MCL1* | *PALB2* | *RAC3* | *SOX21* | *WRN* |
| *BAI3* | *CSF1R* | *ETV6* | *HLA-A* | *MDM2* | *PALLD* | *RAD50* | *SPOP* | *WT1* |
| *BAK1* | *CTCF* | *EWSR1* | *HNF1A* | *MDM4* | *PARK2* | *RAD51* | *SPRY4* | *XPA* |
| *BAP1* | *CTLA4* | *EXT1* | *HNF1B* | *MECOM* | *PARP1* | *RAD51B* | *SRC* | *XPC* |
| *BARD1* | *CTNNB1* | *EXT2* | *HRAS* | *MED12* | *PARP2* | *RAD51C* | *SRY* | *XRCC1* |
| *BAX* | *CUL3* | *EZH2* | *HSD3B1* | *MEF2B* | *PAX5* | *RAD51D* | *STAG2* | *YAP1* |
| *BCL2* | *CUX1* | *FANCA* | *IDH1* | *MEN1* | *PBRM1* | *RAD54L* | *STAT3* | *ZNF2* |
| *BCL2L11* | *CXCR4* | *FANCC* | *IDH2* | *MET* | *PDCD1* | *RAF1* | *STK11* | *ZNF217* |
| *BCR* | *CYLD* | *FANCD2* | *IFNG* | *MGMT* | *PDCD1LG2* | *RARA* | *STMN1* | *ZNF703* |
| *BIRC3* | *CYP19A1* | *FANCE* | *IFNGR1* | *MITF* | *PDE11A* | *RARG* | *STT3A* |  |
| *BLM* | *CYP2A13* | *FANCF* | *IGF1R* | *MLH1* | *PDGFRA* | *RASGEF1A* | *SUFU* |  |
| *BMPR1A* | *CYP2A6* | *FANCG* | *IGF2* | *MLH3* | *PDGFRB* | *RB1* | *TAP1* |  |
| *BRAF* | *CYP2A7* | *FANCI* | *IKBKE* | *MLLT1* | *PDK1* | *RECQL4* | *TAP2* |  |
| *BRCA1* | *CYP2B6*6* | *FANCL* | *IKZF1* | *MLLT3* | *PGR* | *RELN* | *TEK* |  |
| *BRCA2* | *CYP2C19*2* | *FANCM* | *IL7R* | *MLLT4* | *PHOX2B* | *RET* | *TEKT4* |  |
| *BRD4* | *CYP2C9*3* | *FAT1* | *INPP4B* | *MPL* | *PIK3C3* | *RHOA* | *TERC* |  |

NGS, next-generation sequencing.

Table S2. Association analysis of clinical factors and four main driver genes mutation in tumor tissue with survival in 40 metastatic PAC patients

| Variables | No. | OS | | |  | PFS | | |
| --- | --- | --- | --- | --- | --- | --- | --- | --- |
|  |  | OS(m) | 95%CI | *p* |  | PFS(m) | 95%CI | *p* |
| **Age** |  |  |  | 0.849 |  |  |  | 0.426 |
| ＜60 | 22 | 9.1 | 6.6-11.7 |  |  | 4.4 | 3.6-5.2 |  |
| ≥60 | 18 | 9.4 | 6.8-11.9 |  |  | 5.4 | 4.5-6.4 |  |
| **Sex** |  |  |  | 0.074 |  |  |  | 0.034 |
| Male | 29 | 8.7 | 6.8-10.7 |  |  | 4.7 | 4.0-5.3 |  |
| Female | 11 | 12.6 | 8.7-16.4 |  |  | 6.1 | 4.3-8.0 |  |
| **ECOG PS** |  |  |  | ＜0.001 |  |  |  | 0.043 |
| 0-1 | 28 | 10.3 | 6.3-14.3 |  |  | 4.9 | 4.6-5.3 |  |
| 2 | 12 | 4.1 | 1.8-6.4 |  |  | 4.1 | 0-8.4 |  |
| **Primary tumor location** |  |  |  | 0.823 |  |  |  | 0.911 |
| Head or Neck | 13 | 10.1 | 1.6-18.5 |  |  | 5.3 | 3.4-7.1 |  |
| Body or Tail | 27 | 9.1 | 7.3-11.0 |  |  | 4.8 | 4.3-5.2 |  |
| **Grade** |  |  |  | 0.179 |  |  |  | 0.335 |
| Low | 23 | 8.7 | 6.2-11.3 |  |  | 4.8 | 3.7-5.8 |  |
| Middle or High | 17 | 10.1 | 4.1-16.0 |  |  | 5.4 | 4.1-6.7 |  |
| **Metastatic organ** |  |  |  | 0.211 |  |  |  | 0.052 |
| One | 17 | 8.1 | 4.7-11.4 |  |  | 4.9 | 3.7-6.1 |  |
| Multiple | 23 | 10.1 | 8.6-11.5 |  |  | 5.4 | 3.5-7.3 |  |
| **Metastatic lesion location** |  |  |  | 0.706 |  |  |  | 0.303 |
| Hepatic metastasis | 34 | 9.1 | 7.3-11.0 |  |  | 5.0 | 4.3-5.6 |  |
| Non-hepatic metastasis | 6 | 10.3 | 0-24.5 |  |  | 4.3 | 1.9-6.6 |  |
| **CA-199(u/mL)** |  |  |  | <0.001 |  |  |  | 0.010 |
| ≤3851 | 20 | 14.3 | 10.4-18.3 |  |  | 5.3 | 3.6-6.9 |  |
| ＞3851 | 20 | 5.9 | 3.0-8.7 |  |  | 3.8 | 2.6-5.0 |  |
| **CEA(μg/l)** |  |  |  | 0.004 |  |  |  | 0.012 |
| ≤8.9 | 20 | 12.9 | 2.9-22.8 |  |  | 5.7 | 3.3-8.2 |  |
| ＞8.9 | 20 | 7.5 | 3.0-12.1 |  |  | 4.4 | 3.8-4.9 |  |
| **NLR** |  |  |  | 0.007 |  |  |  | 0.05 |
| ≤2.9 | 20 | 12.6 | 6.4-18.7 |  |  | 5.3 | 2.8-7.7 |  |
| ＞2.9 | 20 | 7.5 | 5.4-9.7 |  |  | 4.3 | 3.3-5.3 |  |
| **PLR** |  |  |  | 0.021 |  |  |  | 0.308 |
| ≤140.1 | 20 | 10.3 | 1.1-19.4 |  |  | 5.0 | 4.5-5.5 |  |
| ＞140.1 | 20 | 7.7 | 3.2-12.3 |  |  | 4.5 | 2.0-7.1 |  |
| ***KRAS*** |  |  |  | 0.914 |  |  |  | 0.114 |
| *KRAS* + | 35 | 9.1 | 8.1-12.0 |  |  | 5.3 | 4.2-6.3 |  |
| *KRAS* - | 5 | 10.1 | 6.8-11.5 |  |  | 4.5 | 4.2-4.9 |  |
| ***TP53*** |  |  |  | 0.344 |  |  |  | 0.198 |
| *TP53* + | 30 | 9.1 | 7.8-10.5 |  |  | 5.0 | 4.1-5.9 |  |
| *TP53* - | 10 | 8.0 | 4.4-11.7 |  |  | 4.4 | 3.6-5.1 |  |
| ***CDKN2A*** |  |  |  | 0.687 |  |  |  | 0.528 |
| *CDKN2A* + | 9 | 5.8 | 4.2-7.5 |  |  | 3.5 | 2.0-5.1 |  |
| *CDKN2A* - | 31 | 9.8 | 8.6-11.0 |  |  | 5.0 | 4.3-5.7 |  |
| ***SMAD4*** |  |  |  | 0.152 |  |  |  | 0.22 |
| *SMAD4* + | 7 | 6.7 | 0-13.3 |  |  | 3.8 | 1.8-5.8 |  |
| *SMAD4* - | 33 | 9.8 | 8.5-11.0 |  |  | 5.3 | 4.2-6.3 |  |
| **Combination** |  |  |  | 0.048 |  |  |  | 0.433 |
| 0-2gene+ | 25 | 10.1 | 8.2-12.0 |  |  | 5.3 | 4.5-6.1 |  |
| 3-4gene+ | 15 | 6.7 | 3.9-9.5 |  |  | 4.1 | 2.5-5.6 |  |

OS, overall survival; PFS, progression-free survival; PAC, pancreatic adenocarcinoma; CI, confidence interval; ECOG PS, Eastern Cooperative Oncology Group Performance Status; NLR, neutrophil-lymphocyte ratio; PLR, platelet- lymphocyte ratio.

Table S3. P value of correlation among clinical factors in 40 metastatic PAC

| Variables | CA19-9 | CEA | NLR | PLR |
| --- | --- | --- | --- | --- |
| Age | 0.514 | 0.783 | 0.7 | 0.757 |
| Sex | 0.146 | 0.628 | 0.218 | 0.385 |
| ECOG PS | 0.361 | 0.033 | 0.004 | 0.023 |
| Grade | 0.293 | 0.389 | 0.85 | 0.516 |
| Primary tumor location | 0.516 | 0.859 | 0.113 | 0.458 |
| Metastatic organ | 0.049 | 0.909 | 0.516 | 0.893 |
| Metastatic lesion location | 0.981 | 0.099 | 0.184 | 0.272 |

PAC, pancreatic adenocarcinoma; ctDNA, circulating tumor DNA; ECOG PS, Eastern Cooperative Oncology Group Performance Status; NLR, neutrophil-lymphocyte ratio; PLR, platelet- lymphocyte ratio.

Table S4. Somatic mutation, Germline mutation, and CNV （Copy number variation） of 40 patients

| Patient NO. | Somatic mutation NO. | Germline mutation | CNV |
| --- | --- | --- | --- |
| 1 | 2 | MLH1 gene V384D heterozygous germline mutation | 0 |
| 2 | 6 | 0 | 0 |
| 3 | 5 | 0 | RICTOR gene amplification, TP53 single copy number deletion |
| 4 | 3 | 0 | AKT2 gene amplification, CCNE1 gene amplification |
| 5 | 4 | 0 | 0 |
| 6 | 3 | GATA6-S184N heterozygous germline mutation, PDE11A-R7fs heterozygous germline deletion shift mutation | MYCL gene amplification, SOX2 gene amplification |
| 7 | 2 | RAD51C-R237X heterozygous strain truncation mutation | 0 |
| 8 | 4 | 0 | RB1 single copy number deletion |
| 9 | 5 | GATA5-E51K heterozygous germline mutation | FGFR4 gene amplification, VEGFR3 gene amplification, KRAS gene amplification, PDGFRB gene amplification, RB1 single copy number deletion |
| 10 | 1 | 0 | 0 |
| 11 | 6 | POLE-Q1950X Heterozygous germline truncation mutation | KRAS gene amplification |
| 12 | 6 | 0 | 0 |
| 13 | 2 | 0 | FGFR1 gene amplification, TP53 single copy number deletion, ZNF703 gene amplification |
| 14 | 3 | 0 | MYC gene amplification |
| 15 | 7 | PDE11A-G57fs Heterozygous germline deletion shift mutation | CDK6 gene amplification, HGF gene amplification |
| 16 | 13 | 0 | MDM2 gene amplification |
| 17 | 2 | 0 | 0 |
| 18 | 4 | 0 | 0 |
| 19 | 4 | 0 | 0 |
| 20 | 7 | 0 | CCNE1 gene amplification |
| 21 | 5 | 0 | CDK6 gene amplification |
| 22 | 4 | 0 | 0 |
| 23 | 6 | 0 | HGF gene amplification, TP53 single copy number deletion |
| 24 | 3 | 0 | VEGFA gene amplification |
| 25 | 13 | 0 | HNF1B gene amplification, PIK3CA gene amplification, PRKCI gene amplification, TERC gene amplification, TP53 single copy number deletion |
| 26 | 3 | 0 | 0 |
| 27 | 6 | 0 | FGFR1 gene amplification, GATA4 gene amplification, NOTCH2  gene amplification, ZNF703 gene amplification |
| 28 | 6 | 0 | TP53 single copy number deletion |
| 29 | 6 | 0 | ZNF703 gene amplification |
| 30 | 1 | GATA6-p.S184N exon 2  heterozygous germline mutation | 0 |
| 31 | 3 | 0 | 0 |
| 32 | 5 | 0 | SMARCB1 single copy number deletion, TP53 single copy number deletion |
| 33 | 5 | 0 | AKT3 gene amplification, DDR2 gene amplification, MDM4 gene amplification, NTRK1 gene amplification |
| 34 | 4 | 0 | 0 |
| 35 | 2 | 0 | 0 |
| 36 | 5 | 0 | MCL1 gene amplification |
| 37 | 2 | 0 | 0 |
| 38 | 2 | 0 | GATA6 gene amplification |
| 39 | 9 | 0 | GATA6 gene amplification, MCL1 gene amplification |
| 40 | 5 | 0 | 0 |

Table S5. Concordance of DNA in tumor tissue and plasma in 35 metastatic PAC

| Concordance | Patient number |
| --- | --- |
| Fully concordant | 30 |
| Partial concordant | 5(tissue+/ctDNA- in 4; tissue-/ctDNA+ in 1) |

Table S6. P value of correlation among clinical factors and gene mutation in ctDNA in 35 metastatic PAC

| Variables | KRAS | TP53 | CDKN2A | SMAD4 |
| --- | --- | --- | --- | --- |
| Age | 0.262 | 1.000 | 1.000 | 0.366 |
| Sex | 1.000 | 0.226 | 0.299 | 0.299 |
| ECOG PS | 0.015 | 0.259 | 1.000 | 0.391 |
| Grade | 0.698 | 0.258 | 0.648 | 0.377 |
| Primary tumor location | 1.000 | 0.709 | 0.146 | 0.352 |
| Metastatic organ | 1.000 | 0.467 | 0.680 | 0.367 |
| Metastatic lesion location | 0.095 | 0.026 | 0.561 | 0.561 |

PAC, pancreatic adenocarcinoma; ctDNA, circulating tumor DNA; ECOG PS, Eastern Cooperative Oncology Group Performance Status.
